# Heterozygous *De Novo* Truncating Mutation of Nucleolin in an ASD Individual Disrupts Its Nucleolar Localization

**DOI:** 10.3390/genes13010051

**Published:** 2021-12-24

**Authors:** Taimoor I. Sheikh, Ricardo Harripaul, Nasim Vasli, Majid Ghadami, Susan L. Santangelo, Muhammad Ayub, Roksana Sasanfar, John B. Vincent

**Affiliations:** 1 Molecular Neuropsychiatry & Development (MiND) Lab, Campbell Family Mental Health Research Institute, Centre for Addiction and Mental Health, Toronto, ON M5T 1R8, Canada; Taimoor.Sheikh@nygh.on.ca (T.I.S.); ricardo.harripaul@gmail.com (R.H.); 2 Institute of Medical Science, University of Toronto, Toronto, ON M5S 1A8, Canada; 3 Department of Pediatric Laboratory Medicine, Hospital for Sick Children, Toronto, ON M5G 1X8, Canada; nasim.vasli@hotmail.com; 4 Department of Educational Sciences, Farhangian University, Tehran 19989-63341, Iran; drghadami50@gmail.com; 5 Center for Psychiatric Research, Maine Medical Center Research Institute, Portland, ME 04101, USA; SSantangel@mmc.org; 6 Department of Psychiatry, Tufts University School of Medicine, Boston, MA 02110, USA; 7 Department of Psychiatry, Maine Medical Center, Portland, ME 04102, USA; 8 Department of Psychiatry, Queen’s University, Kingston, ON K7L 7X3, Canada; ma84@queensu.ca; 9 Department of Academic Psychiatry, University College London, London WC1E 6BT, UK; 10 Department of Psychiatry, Harvard Medical School, Boston, MA 02215, USA; roxanasasanfar@gmail.com; 11 Psychiatric and Neurodevelopmental Genetics Unit, Center for Human Genetic Research, Massachusetts General Hospital, Boston, MA 02114, USA; 12 Department of Psychiatry, University of Toronto, Toronto, ON M5T 1R8, Canada

**Keywords:** nucleolin, C23, nucleolar localization signal, autism spectrum disorder (ASD), GAR domain

## Abstract

Nucleolin (NCL/C23; OMIM: 164035) is a major nucleolar protein that plays a critical role in multiple processes, including ribosome assembly and maturation, chromatin decondensation, and pre-rRNA transcription. Due to its diverse functions, nucleolin has frequently been implicated in pathological processes, including cancer and viral infection. We recently identified a *de novo* frameshifting indel mutation of *NCL*, p.Gly664Glufs*70, through whole-exome sequencing of autism spectrum disorder trios. Through the transfection of constructs encoding either a wild-type human nucleolin or a mutant nucleolin with the same C-terminal sequence predicted for the autism proband, and by using co-localization with the nucleophosmin (NPM; B23) protein, we have shown that the nucleolin mutation leads to mislocalization of the NCL protein from the nucleolus to the nucleoplasm. Moreover, a construct with a nonsense mutation at the same residue, p.Gly664*, shows a very similar effect on the location of the NCL protein, thus confirming the presence of a predicted nucleolar location signal in this region of the NCL protein. Real-time fluorescence recovery experiments show significant changes in the kinetics and mobility of mutant NCL protein in the nucleoplasm of HEK293Tcells. Several other studies also report *de novo* *NCL* mutations in ASD or neurodevelopmental disorders. The altered mislocalization and dynamics of mutant NCL (p.G664Glufs*70/p.G664*) may have relevance to the etiopathlogy of *NCL*-related ASD and other neurodevelopmental phenotypes.

## 1. Introduction

Nucleolin (C23; OMIM: 164035) is one of the most abundant proteins in the nucleolus, accounting for almost 10% of the total protein content [1,2]. During interphase, the nucleolus is formed by three components: the granular component (GC), the dense fibrillar component (DFC), and the fibrillar center (FC) [3]. Pre-rRNA early processing takes place in the DFC, whereas the later processing steps, RNA modification and the formation of pre-ribosomal particles, take place in the GC [4]. During mitotic cell division (interphase), the nucleolin protein is localized within the nucleolar DFC and GC [5] and close to the kinetochore of chromosomes; then, from the prometaphase to anaphase stages, it has been observed to be associated with the spindle poles [6]. A depletion of nucleolin protein leads to a disruption in the congression of chromosomes, improper kinetochore attachments, and an effect on the spindle assembly in the HeLa cells [6]. Under certain conditions, nucleolin has also been reported to have been found in the cytoplasm, such as under heat shock, γ-irradiation, and CPT (camptothecin) treatment [7,8].

**Figure 1:**
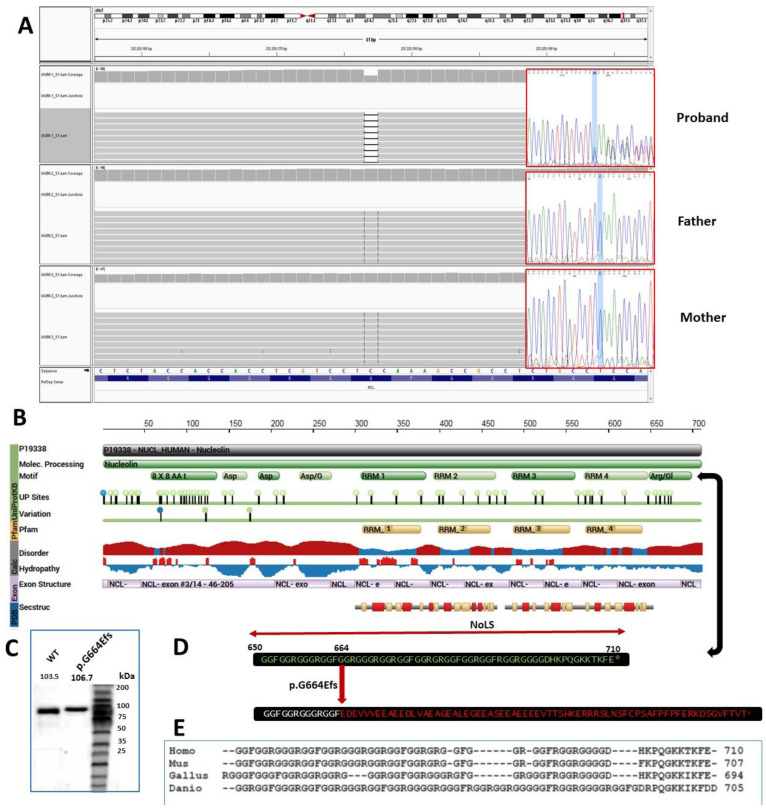
**NCL mutation and protein structure.** (**A**) Alignment of whole-exome sequence reads for proband, father and mother in Integrated Genomics Viewer (IGV: https://software.broadinstitute.org/software/igv/ (accessed on October 2021)), and electropherograms for Sanger sequencing validation (through tcag.ca). (**B**) NCL protein feature view using Interpro (www.ebi.ac.uk/interpro (accessed on October 2021)) showing known and/or predicted domains, post-translational modifications, phosphorylation sites and disorder and hydropathic regions of NCL protein. (**C**) Western blot showing GFP fused full-length wild-type and p.G664Efs recombinant NCL protein. (**D**) C-terminal residues of wild-type and p.G664Efs NCL protein and (**E**) The evolutionary conservation analysis of nucleolin C-terminal (GAR domain) from different species, using ClustalOmega (https://www.ebi.ac.uk/Tools/msa/clustalo/ (accessed on October 2021)), using sequences from human (NP_005372.2), mouse (NP_035010.3), chicken (NP_990596), and zebrafish (*Danio rerio*: NP_001070120.2). RRM = RNA recognition motif and NoLS = Nucleolar Localization Signal.

The nucleolin protein consists of three domains: a disordered N-terminal domain containing several acidic regions and phosphorylation sites; four RNA recognition motifs (RRM) or RNA binding domains (RBD) within the central region; and a C-terminal Gly/Arg-rich (GAR) or Arg-Gly-Gly (RGG) domain with several glycine/arginine-rich motifs (Figure 1B) [9]. The sequence comparison analysis of nucleolin C-terminal (GAR domain) from different species revealed a high degree of evolutionary conservation (Figure 1E).

Among the known RNA binding proteins, nucleolin is one of the most multifunctional proteins. The RRMs of nucleolin interact with the stem-loop structure of RNA, and are known to be involved in the modification and processing of pre-rRNA [10]. The C-terminal GAR domains are involved in the ribosomal assembly and nuclear import of ribosomal proteins [10]. The RRM4 (or RBD4) and the C-terminal GAR/RGG domain of nucleolin binds to the human telomerase reverse transcriptase subunit (hTERT), and this interaction also involves the telomerase RNA subunit hTERC, which is critical for the nucleolar localization of hTERT [11]. Several reports have shown the critical role of the (GAR) domain in nucleolar localization independent of methylation [12,13]. Here, we report the follow-up functional studies of a novel frameshift mutation, p.Gly664Glufs*70, identified as *de novo* in an individual presenting with autism spectrum disorder (ASD), which replaced the last 47 C-terminal GAR residues of nucleolin protein with 69 non-GAR residues (Figure 1D).

## 2. Materials and Methods

### 2.1. Subject Ascertainment and Assessment

Institutional Research Ethics Board approval was received for this study through the Centre for Addiction and Mental Health (CAMH) and the Special Education Organization (SEO), Iran. The IAU66 proband/father/mother trio was ascertained by Dr. Sasanfar by the Children’s Health and Evaluation project (CHEP), sponsored by the Special Education Organization (SEO), as described previously [14]. The diagnostic assessment for ASD was performed using an ADI-R and ADOS assessment by certified clinicians, and diagnosed by a child psychiatrist [14].

### 2.2. Variant Identification and Annotation Pipeline

Whole-exome sequencing of the male ASD individual and both parents was conducted using Illumina HiSeq 2500 (Illumina Inc., San Diego, CA, USA). The mutation was confirmed by Sanger sequencing (The Center for Applied Genomics, www.tcag.ca (accessed on September 2021)). For details on the DNA library preparation and sequencing, as well as the sequence alignment and analysis pipeline, see [15]. The comparison of proband and parental exome sequence variants for the identification of *de novo* variants was performed as described in Harripaul et al. (MedRxiv and Ph.D. thesis) [16,17].

### 2.3. Cloning, Mutagenesis, Cell Culture and Transfections

HEK293T human embryonic kidney cells (ATCC CRL-3216TM) were used for the various localization experiments. Full-length NCL (NM_005381.3) was cloned into the mammalian expression vector pDEST47, C-terminally GFP-tagged using the Gateway^TM^ cloning system (ThermoFisher scientific, Waltham, MA, United States). Site-directed mutagenesis was used to generate a p.Gly664Glufs mutation NCL construct, using the forward NCL-G664Fs-F GGCCGCGGCGGCTTTGAGGACGAGGTGGTG GTA and reverse NCL-G664Fs-R TACCACCACCTCGTCCTCAAAGCCGCCGCGGCC, and a p.Gly664* mutation construct, using forward NCL-G664del-F GGCAGAGGCGGCTTTTGAGGACGAGGTGGTG and reverse

NCL-G664del-R CACCACCTCGTCCTCAAAAGCCGCCTCTGCC. All mutations were introduced through PCR-based site-directed mutagenesis, according to the manufacturer’s instructions (Quikchange Lightning site-directed mutagenesis kit, Agilent Technologies, Santa Clara, CA, USA). To transfect WT and Mut fusion proteins in HEK293T cells, either PolyFect (Qiagen, Hilden, Germany) or polyethylenimine based transfection reagents were used (jetPEI^®^, PolyPlus, Illkirch, France), following the manufacturers’ instructions.

### 2.4. Immunofluorescence (IF) and DNA Staining

All the transfected cells were fixed 48 h post-transfection, using 4% paraformaldehyde, followed by 2X washes with phosphate-buffered saline (PBS). Transfected HEK293T cells were permeabilized with PBS (Tween-20, 0.05%) and blocked in 10% goat serum for 2 h. Immunofluorescence staining, using monoclonal anti-B23 (Nucleophosmin/NPM) (1:500 Cat #B0556; Sigma-Aldrich, St. Louis, MO, USA), was conducted overnight, followed by a Cy3-conjugated goat anti-mouse IgG antibody (1:800 Cat#AP124C; EMD Millipore, Billerica, MA, USA). The cells were then counterstained with DAPI and visualized under a confocal fluorescence microscope.

### 2.5. Fluorescence Confocal Microscopy

Z-stack images acquired with a frame size of 512 × 512 pixels were used to study NCL (C23) and B23 co-localization. A confocal microscope, Olympus FV1200, using 100X/1.3 NA oil objective, was used to capture DAPI (heterochromatin) at 405 nm, 488 nm for GFP (recombinant NCL-GFP fusion protein), and 588nm for mCherry (B23-Cy3 conjugate).

### 2.6. Fluorescence Recovery after Photobleaching (FRAP)

Exogenous NCL was expressed in HEK293T cells in chambered cover glass culture plates (Nunc™; NalgeNunc, Rochester, NY, USA). To capture the recovery and diffusion dynamics, FRAP assays were used. All FRAP experiments were performed in temperature (37 °C)- and CO_2_ (5%)-controlled incubation chambers in the HEK293T cells in 10 independent FRAP assays. In all time-lapse imaging, a series of confocal time-lapse images of frames (512 × 512 pixels) was captured at a 488 nm laser excitation with 0.05 transmissions for GFP-tagged protein, post-bleach recovery. All the FRAP assay images were recorded with a minimum of 10 pre-bleach frames, 250 µs bleach time, with a 405 nm laser line at 100% transmission, and 150 post-bleach frames were recorded at equal time intervals.

## 3. Results

### 3.1. Mutation Identification and Annotation

The mutation (chr2:232320177_232320177delC; NM_005381.3:c.1991delG; p.Gly664Glufs*70) was identified by the Whole-Exome Sequencing (WES) of a set of ASD trios, as described elsewhere [16,17], and after verification, using Sanger sequencing, in the ASD individual and both biological parents, this mutation turned out as de novo (Figure 1A). The Gly664Glufs*70 mutation is not present in the gnomAD control database v2.1.1 (251,378 alleles, including 208,110 from the non-neuro subset). The mutation lies within the penultimate exon of the gene *NCL*, encoding the nucleolin protein; however, despite causing a frameshift, this does not result in a premature stop codon, and thus, the nonsense-mediated mRNA decay is not predicted (using www.mutationtaster.org, accessed on September 2021). The p.Gly664Glufs*70 mutation replaces the last 47 C-terminal residues of nucleolin protein.

(GGRGGGRGGRGGFGGRGRGGFGGRGGFRGGRGGGGDHKPQGKKTKFE) with 69 non-GAR residues (EDEVVVEEAEEDLVAEAGEALEGEEASEEAEEEEVTTSHKERRRSLNSFCPSAFPFPFERKDSGVFTVT) (Figure 1D). The computational physical and chemical parameters of the wild-type residues using the ProtParam tool (https://web.expasy.org/protparam/ accessed on October 2021) [18] indicate the following predicted changes in the NCL GAR domain due to the p.G663fs mutation: (1) molecular weight: WT (4502.91) vs. Mutant (7708.19); (2) theoretical pI: WT (12.23) vs. Mutant (4.08); (3) Arg composition: WT (17.0%) vs. Mutant (5.8%); (4) Gly composition: WT (53.2%) vs. Mutant (4.3%); (5) instability index: WT (35.19, which classifies the protein as stable) vs. Mutant (92.60, which classifies the protein as unstable); (6) aliphatic index: WT (0.00) vs. Mutant (56.52); and (7) grand average of hydropathicity (GRAVY): WT (−1.413) vs. Mutant (−0.771).

### 3.2. NCL p.Gly664Glufs*70 Disrupts Nucleolar Localization

**Figure 2:**
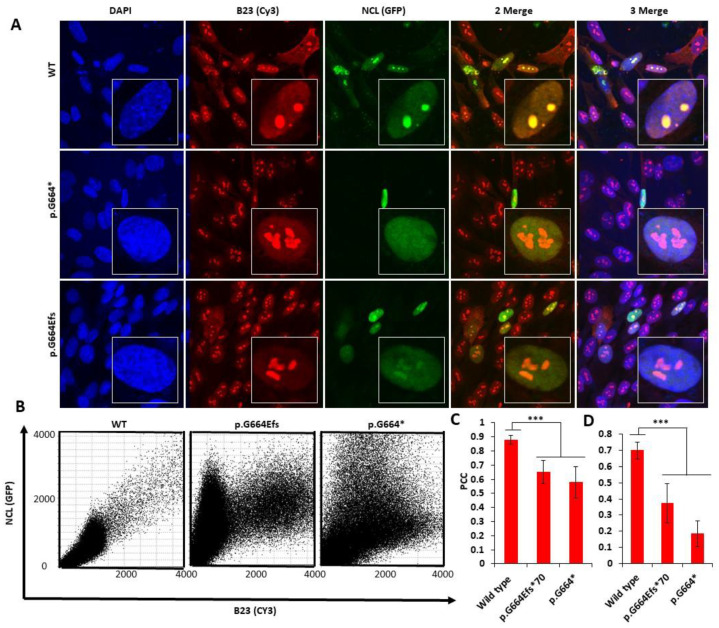
**Comparison of the nucleolar localization of wild-type and mutant NCL expressing in HEK293T cells.** (**A**) Confocal image stacks representing (left to right): column 1 DAPI (Blue), column 2 B23 (Red—CY3), column 3 NCL (green—GFP), column 4 merge B23-NCL CY3 (red) and GFP (green), column 5 All merge DAPI-GFP-Red (DNA-NCL-B23). Row 1 (left to right) wild-type, Row 2 (left to right) p.G664*, and Row 3 (left to right) p.G664Efs*70. Note that all images shown here are representative of the authors’ observations; averaged data from all images are presented in the quantitative analysis and discussed in the main text. (**B**) Scatter plot of red and green pixel intensities showing co-localization of B23 (Red—Nucleolus) and recombinant NCL protein (GFP). (**C**) Pearson’s correlation coefficient (PCC) values of B23 (Red—Nucleolus) and recombinant NCL protein (GFP) were plotted to represent quantitative co-localization of NCL in nucleus and (**D**) Nucleolus (n^WT^ = 14, n^p.G664^ = 16 and n^p.G664Efs^ = 16; *p* *** values ≤ 0.005 one-way ANOVA; ±SD is shown. Additional co-localization images are shown in Supplementary Material Figure S1.

To study the effect of p.Gly664Glufs*70 on the localization of the NCL protein, N-terminally GFP-tagged WT full-length and mutant truncated NCL proteins were transiently expressed in HEK293T cells. A slightly larger protein showing the insertion of few residues caused by this mutation was shown by anti-GFP western blots of protein extracted from the cell lysate expression recombinant NCL proteins (Figure 1C). The cells were counterstained with DAPI to visualize the nucleus and an anti-B23 antibody along with a Cy3-labelled secondary anti-IgG antibody to counterstain the nucleolus (Figure 2). The co-localization analysis of WT vs. p.Gly664Glufs*70, as well as WT vs. p.Gly664*, revealed significant disruption of the expected localization of the NCL protein into the nucleolus (Figure 2C). To investigate the effect of a complete deletion of GAR residues, we analyzed the localization of a deletion construct and found a complete disruption of the nucleolar localization of the NCL protein (Figure 2B).

### 3.3. NCL p.G664Efs*70 Affects Protein Mobility and Dynamics

**Figure 3:**
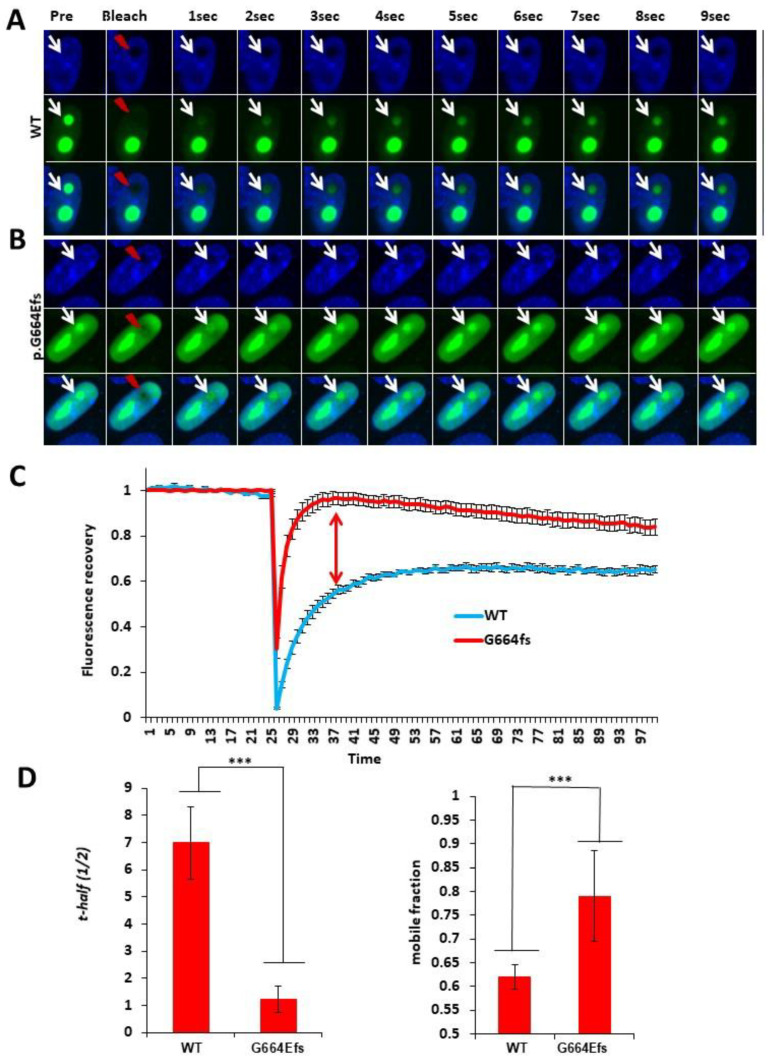
Nucleolar FRAP experiments to analyze mobility and binding dynamics of wild-type and mutant NCL protein in HEK293T cells. Real-time post-bleach recovery of GFP tagged recombinant NCL protein were captured after bleaching NCL localized in the nucleolus (unstained Hoechst 33342 regions) at ~200 µs. Red ‘lightning’ indicates bleach spots. Rows (left to right): Representation of FRAP pre-bleach, bleach and post-bleach real-time recovery of NCL (**A**) wild-type and (**B**) p.G664Efs*70, respectively. Column (Left→Right): column 1 is representing preceding pre-bleach images; column 2 representing bleach images; column 3 representing 1st images; column 4 representing 2nd images, column 5 representing 3rd images and column 11 representing 9th images following bleach, where 1 frame = ~108.784 m.s. (**C**) FRAP recovery curves normalized to 1, showing NCL protein recovery in 100 post bleach frames. (**D**) Comparative illustration of half-maximal recovery time (t-half (1/2)) and mobile fraction of wild-type and p.G664Efs*70 from individual FRAP experiments (*n* = 10 each), *** *p* ≤ 0.05 two-tailed Mann–Whitney non parametric U test. ± S.E. bars are shown.

As the RGG domain of NCL protein is believed to be involved in various steps of the ribosome biogenesis, including RNA polymerase (Pol) I transcription and the processing of pre-rRNA, and the assembly and nucleocytoplasmic transport of ribosome particles [19], we analyzed the effect of p.G663fs on the mobility dynamics using live cell fluorescence recovery after photobleaching (FRAP) assays (Figure 3A,B). The photobleaching of the NCL protein accumulated in the nucleolus followed by real-time recovery imaging for the WT vs. p.G664Efs*70 NCL revealed a highly significant difference in the recovery curve, half-time (t-1/2) WT = 6.99 ± 1.34 vs. p.G664Efs*70 = 1.22 ± 0.48 and mobile fraction WT = 0.62 ± 0.025 and p.G667fs = 0.79 ± 0.095 (Figure 3C,D).

## 4. Discussion

Gene-disrupting de novo mutations have become a common theme in the genetics of autism spectrum disorder [20]. In this study, we report on a frameshift *de novo* mutation in the *NCL* gene. Interestingly, instead of truncating the C-terminal of the NCL protein, this mutation replaces the last 47 C-terminus with 69 non-GAR residues. These substitutions are predicted to have a significant effect on the important chemical properties of the C-terminal region of the NCL protein, such as molecular weight, theoretical pI, Arg and Gly composition, instability index, aliphatic index and GRAVY. The WT residue composition clearly indicates a neutral-positive residue motif (Gly-Arg) (GGRGGGRGGRGGFGGRGRGGFGGRGGFRGGRGGGGDHKPQGKKTKFE), whereas the Mut C-terminus mostly consists of neutral-negative residues (Val/Ala-Glu/Asp) (EDEVVVEEAEEDLVAEAGEALEGEEASEEAEEEEVTTSHKERRRSLNSFCPSAFPFPFERKDSGVFTVT).

The presence of positively charged residues in the experimentally validated nucleolar localization sequences (NoLSs) dataset of different nucleolar protein highlights the importance of the arginine residues at the C-terminus of NCL [21]. Our nucleolar localization studies show supporting evidence for the NoLS of the NCL protein (Figure 2). By using highly sensitive live cell imaging, we have also demonstrated the effect of these changes on the dynamics of the NCL protein (Figure 3).

In summary, by using co-localization and FRAP analyses, we have shown that the NCL p.G664Efs*70 variant causes disruption of the nucleolar localization signal of the NCL protein. The localization of the NCL protein to the nucleolus is critical, as its known functions are restricted to involvement with the nucleolar machinery; therefore, mislocalization is likely to equate with a loss of function.

The FRAP data show altered binding and mobility dynamics of the recombinant NCL p.G664Efs*70 protein. The slow recovery and lower mobile fraction of the wild-type protein indicates the normal mobility and binding of the wild-type NCL protein in the nucleolus, whereas faster mobility and more protein in the mobile fraction indicates more unbound non-functional protein, i.e., more of the protein failed to reach the nucleolus to perform its function (Figure 3C,D).

It should also be noted that the mutation, which is in the penultimate exon of *NCL*, is not predicted to lead to nonsense-mediated mRNA decay (NMD), according to mutationtaster.org. However, since it was not possible to transport viable mRNA or cells from Iran, it was not possible to validate this prediction in vitro.

Several additional de novo mutations in nucleolin have been identified and reported through large genome or exome sequencing studies. In the Deciphering Developmental Disorders Study [22], two patients were identified, both with *de novo* nonsense mutations in *NCL*. One mutation, Chr2:232321342G>A; NM_005381.3: c.1705C>T; p.(Gln569*), was reported for a female subject described as having global developmental delay, a thyroglossal cyst, obesity, short stature, attention deficit hyperactivity disorder, localized hirsutism, deep set eyes, low-set ears, large fleshy ears, dental crowding, bilateral single transverse palmar creases, short tapering fingers, short, broad feet, and small nails. The second mutation, Chr2:232322432G>A, NM_005381.3:c.1369C>T, p.(Arg457*), was reported for an individual described as having ‘abnormality of head or neck, abnormality of musculoskeletal system, abnormality of the nervous system, and growth abnormality. It should be noted that, in addition to the predicted loss of the NCL C-terminus, these mutations result in the loss of one or more of the RRMs. Additionally, these mutations would be predicted to lead to nonsense-mediated mRNA decay (using www.mutationtaster.org (accessed on October 2021)), and hence, possibly more severe consequences than for the mutation we report here, as both the mRNA and protein would be affected (and the more functional domains of the protein would be lost). Two individuals diagnosed with autism spectrum disorder were reported as having *de novo* loss-of-function mutations in *NCL* [23]; however, we have been unable to identify the mutation annotations for these directly.

In population databases for whole-exome and genome sequence data such as gnomAD and ExAC, loss of function (LoF) variants are significantly underrepresented (pLI = 1.0; observed/expected = 2/35.6 = 0.056, in the gnomAD non-neuro control set (N = 114,704)). In the most recent gnomAD release (as of November 2021), v3.1.2 non-neuro controls (~135,000 alleles), there are just three potential LoF variants identified: (1) A splice acceptor variant (chr2:232325005C>T; NM_005381.2:c.899-1G>A; MAF = 7.421 × 10^−6^) at the intron 5/exon 6 junction. If this were to result in removal of exon 6, the resulting transcript would remain in-frame; (2) A splice donor variant (chr2:232321995_232322004del) that only affects a non-canonical exon (present in only one of 11 listed transcripts, namely, ENST00000356936.6); (3) A stop gain just 20 codons ahead of the stop codon in the terminal exon, p.Arg691* (chr2:232319964G>A; NM_005381.2:c.2071C>T; MAF = 7.43 × 10^−6^), which would remove the C-terminal 20 amino acids, but leaving the majority of the GAR or RGG domain intact, and, as with the mutation identified in the proband, is not predicted to trigger NMD. The gnomAD v3.1.2 dataset also lists 12 frameshifting indels; however, upon close inspection, these are in a region listed as a “low complexity region, variant annotation or quality dubious”, and they all pair-off with each other in the individual reads to give non-frameshifting variants.

In contrast, constraint measures for missense and nonsynonymous *NCL* variants are non-significant, with close-to-expected numbers observed. The identification of molecular pathways to link NCL protein with autism or ASD phenotype is yet to be elucidated. However, nucleolin has been shown to interact with the death domain of the p53-induced protein death domain (PIDD) protein 1 [24]. Recently, we, and others, have reported homozygous *PIDD1* mutations segregating in autosomal recessive non-syndromic intellectual disability [15,25,26,27]. Mutations in the gene encoding the PIDD-interactor, CRADD, have also been linked to autosomal recessive intellectual disability and lissencephaly [28]. In addition, direct interaction with NCL has been reported with the Fragile X mental Retardation Protein (FMRP) [29]. Mutations in the Rett syndrome gene *MECP2* have also been shown to disrupt nucleolin-*rRNA*-mTOR-P70S6K signaling [30]. In addition, *NCL* has been reported as a susceptibility gene associated with bipolar disorder [31]. Our results, together with previous observations, provide evidence of a strong link between *NCL* loss-of-function variants and ASD, and we note shared biological pathways with a number of other proteins that have been associated with ASD and other neurodevelopmental disorders.

## Supplementary Materials

The following are available online at https://www.mdpi.com/article/10.3390/genes13010051/s1, Figure S1. Co-localization of exogenous NCL-GFP (wild type (WT), and mutants (p.G664Efs and p.G664*) with endogenous nucleolar protein B23 (Cy3), after transfection in HEK293T cells.
