# Supplementary material for: Heterozygous De Novo Truncating Mutation of Nucleolin in an ASD Individual Disrupts Its Nucleolar Localization
Source: Genes (Basel). 2021 Dec 24;13(1):51. doi: 10.3390/genes13010051 (PMC8774667; doi:10.3390/genes13010051)

Supplementary Materials:

Figure S1. Co-localization of exogenous NCL-GFP (wild type (WT), and mutants (p.G664Efs and p.G664*) with endogenous nucleolar protein B23 (Cy3), after transfection in HEK293T cells. Cells were probed with anti-B23 antibody along with Cy3-labelled secondary anti-IgG antibody.

Supplementary Figure S1

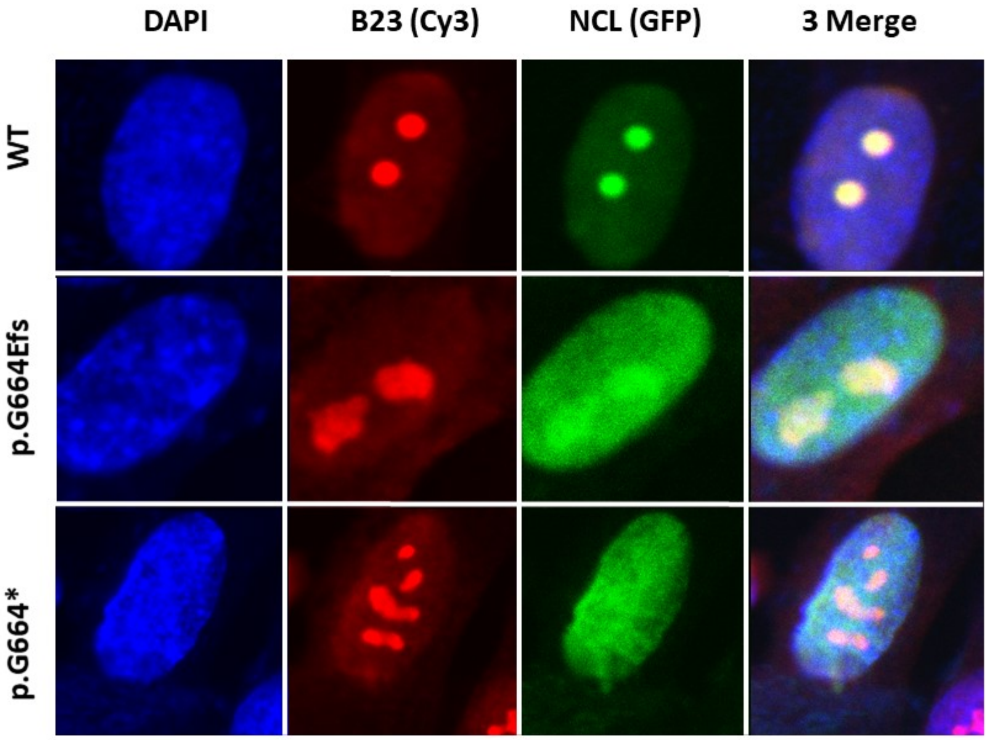

Supplement: Supplementary file 1 [file genes-13-00051-s001.zip › genes-1444392-supplementary.pdf]
